# Comprehensive analysis of *RET *common and rare variants in a series of Spanish Hirschsprung patients confirms a synergistic effect of both kinds of events

**DOI:** 10.1186/1471-2350-12-138

**Published:** 2011-10-13

**Authors:** Rocio Núñez-Torres, Raquel M Fernández, Manuel Jesus Acosta, Maria del Valle Enguix-Riego, Martina Marbá, Juan Carlos de Agustín, Luis Castaño, Guillermo Antiñolo, Salud Borrego

**Affiliations:** 1 Unidad de Gestión Clínica de Genética, Reproducción y Medicina Fetal. Instituto de Biomedicina de Sevilla (IBIS), Hospital Universitario Virgen del Rocío/CSIC/Universidad de Sevilla, Sevilla, Spain; 2 Centro de Investigación Biomédica en Red de Enfermedades Raras (CIBERER), Sevilla, Spain; 3 Departamento de Genómica y Bioinformática. Centro de Investigación Príncipe Felipe (CIPF), Valencia, Spain; 4 Unidad de Gestión Clínica de Cirugía Infantil, Hospital Universitario Virgen del Rocío, Sevilla, Spain; 5 Grupo de investigación en Endocrinología y Diabetes, Hospital de Cruces, Vizcaya, Spain

## Abstract

**Background:**

*RET *is the major gene associated to Hirschsprung disease (HSCR) with differential contributions of its rare and common, coding and noncoding mutations to the multifactorial nature of this pathology. In the present study, we have performed a comprehensive study of our HSCR series evaluating the involvement of both *RET *rare variants (RVs) and common variants (CVs) in the context of the disease.

**Methods:**

*RET *mutational screening was performed by dHPLC and direct sequencing for the identification of RVs. In addition Taqman technology was applied for the genotyping of 3 *RET *CVs previously associated to HSCR, including a variant lying in an enhancer domain within *RET *intron 1 (rs2435357). Statistical analyses were performed using the SPSS v.17.0 to analyze the distribution of the variants.

**Results:**

Our results confirm the strongest association to HSCR for the "enhancer" variant, and demonstrate a significantly higher impact of it in male *versus *female patients. Integration of the *RET *RVs and CVs analysis showed that in 91.66% of cases with both kinds of mutational events, the enhancer allele is in *trans *with the allele bearing the *RET *RV.

**Conclusions:**

A gender effect exists on both the transmission and distribution of rare coding and common HSCR causing mutations. In addition, these *RET *CVs and RVs seem to act in a synergistic way leading to HSCR phenotype.

## Background

Hirschsprung disease (HSCR, OMIM #142623) is a congenital malformation of the hindgut produced by a disruption in the neural crest cells (NCC) migration during embryonic development. This disorder results in an absence of intramural ganglion cells in the submucosal and myenteric plexuses producing a functional intestinal obstruction. According to the aganglionic segment length, patients could be classified as short-segment HSCR (S-HSCR), when aganglionosis extend as far as the rectosigmoid junction, and long-segment HSCR (L-HSCR), when it extends beyond it. HSCR presents an estimated incidence of 1/5000 live births with sex-dependent penetrance and male predominance of 4:1 [1,2]. It most commonly presents sporadically, although it can also be familial (up to 20% of the cases). The disease usually presents as isolated, though 30% of the cases are associated with chromosomal abnormalities, neurodevelopmental disorders and a variety of additional isolated anomalies and syndromes [2].

HSCR has a complex genetic etiology with several genes being described as associated with either isolated or syndromic forms. These genes encode for receptors, ligands (especially those participating in the *RET *and *EDNRB *signaling transduction pathways), transcriptional factors or other cell elements that are usually involved in the NCC development and migration that give rise to the enteric nervous system (ENS). Nevertheless, the *RET *proto-oncogene (OMIM +164761) is considered the major disease causing gene in HSCR [2].

*RET *has been extensively studied in HSCR patients and over 100 mutations have been identified along the gene (see HGMD). However, mutations in the *RET *coding sequence (CDS) account for only up to 50% or 7-20% of familial and sporadic cases, respectively [2]. The involvement of *RET *in the pathogenesis of HSCR is further supported by the existence of a specific haplotype, constituted by common *RET *polymorphisms, which seems to be responsible for the majority of sporadic forms [3,4]. This HSCR-associated *RET *haplotype is characterized by a common allele (c.73+9277T, rs2435357) within a conserved enhancer-like sequence in intron 1 (MCS+9.7) [3,5], making a 20-fold greater contribution to risk than coding mutations do [3]. It has been demonstrated a difference in ability of SOX10 to bind to MCS+9.7 and transactivate *RET *depending on the bearing allele at such specific locus [5]. Recently, one SNP located closed to rs2435357 and in complete linkage disequilibrium with it (c.73+9494A > C, rs2506004) has been identified as a binding site for NXF/ARNT2 and SIM2-ARNT2 that modifies *RET *expression, demonstrating that more than one SNP can influence gene expression and ultimately HSCR phenotype [6]. In this way, the combination of common variants (CV), such as the so-called enhancer variants, and rare variants (RV), such as *RET *CDS mutations, seems to explain in part the complexity of HSCR.

In the present study, we have evaluated the implication of both *RET *CVs and RVs in our whole cohort of 282 Spanish patients. In order to elucidate the molecular basis of the sex-dependent difference in HSCR we sought to perform a case-control study to analyze the distribution of the *RET *variants based in gender. In addition, we have performed a segregation analysis in patients for both RVs and CVs, with the aim to clarify their contribution to HSCR phenotype.

## Methods

### Patients and Control Subjects

In the mutational screening we have included an extension of our previously described cohort [7] incorporating a total of 176 new Spanish HSCR patients. 170 of these patients were sporadic cases, 6 were familial belonging to 3 different families. Taking together all the data, our cohort is constituted by a total of 282 HSCR patients (253 sporadic and 29 familial cases belonging to 16 families). According to the length of the aganglionic segment, the patients were catalogued as S-HSCR (55,08%), L-HSCR (11,58%) and Total Colonic Aganglionosis (TCA) (5,61%), while this information was not available for 27,73% of HSCR patients. Male:female ratios were 4:1 and 3:1 for short and long forms (including the TCA patients), respectively. 4,6% of our HSCR patients were syndromic cases with Down syndrome (6 cases), Waardenburg syndrome (2 cases), Ondine course (1 case), cardiopathy (2 cases) or encephalopathy (2 cases). However due to incomplete clinical data of part of our patients, our percentage might be underestimated, taking into account that the overall percentage reported for syndromic HSCR patients is around 18% [2].

Genotyping analysis of *RET *CVs was performed exclusively in the 253 sporadic cases of our cohort, since previous studies suggest that CVs have a minor effect in familial cases, being those cases more probably associated to CDS mutations in *RET *or other secondary genes [2]. 103 of our sporadic cases had been already evaluated in a previous study [8], thus we have genotyped the remaining 150 sporadic cases, as well as their available parents. Trios necessary for the Transmission Disequilibrium Test (TDT) analysis could be completed in 134 of the 150 new families.

A total of 178 control individuals (129 males and 49 females) comprising unselected, unrelated, race, age, and sex-matched individuals were included in this study. All the controls were healthy voluntary donors, who came to the Hospital for other reasons and did not present any symptom suggestive of HSCR.

An informed consent was obtained from all the participants for clinical and molecular genetic studies. The study was approved by the Ethics Committee for clinical research in the Hospital Universitario Virgen del Rocio of Seville, and complies with the tenets of the declaration of Helsinki.

### PCR, dHPLC and Sequence analysis

We screened all the *RET *exons, including intron/exon boundaries by PCR-dHPLC as previously described [7]. The exons of those patients with aberrant wave profiles were subjected to sequence analysis as previously reported [9]. When a novel *RET *variant was identified in a proband, all the available family members were analyzed for the appropriate exon in order to perform the corresponding segregation analysis.

### Genotyping of *RET *SNPs and statistical analysis

We sought to analyze the distribution of three *RET *SNPs (rs2435357 (c.73+9277T > C), rs2505532 (c.74-1370C > T) and rs2565206 (c.74-126G > T)) in HSCR patients to compare with that of the controls obtained in our previous studies [4,8]. Large scale genotyping of all these *RET *SNPs was performed using TaqMan-based techniques for allelic discrimination (TaqMan^® ^SNP Genotyping Assay, Applied Biosystems, Foster City, CA) according to the manufacturer's recommendations and described elsewhere [10].

For each variant, Hardy-Weinberg equilibrium was verified in both the control and patients groups. Allelic and genotypic frequencies of the 3 *RET *SNPs were calculated and then compared between patients and controls. We also compared transmitted *versus *non-transmitted alleles from unaffected parents to affected offspring for each locus. On the other hand, *RET *haplotypes were constructed as previously described [4,8], and their distribution was compared between patients and controls as well as between transmitted and non-transmitted chromosomes in the context of the HSCR trios. Furthermore, allelic, genotypic and haplotypic distributions were compared between cases and controls in different subsets based in gender (male patients *versus *male controls, and female patients *versus *female controls). Despite the fact that the group of male patients is higher that the group of females, each subset of patients was compared to a group of sex-matched controls similar in size. All the comparisons were analysed using χ^2 ^test with Yate's correction, considering statistical significance at p < 0.05 and 2 degrees of freedom (SPSS v.17.0 and R-software). For each variant we calculated the OR (95% CI) for the recessive, dominant and additive models (unadjusted and adjusted for gender) [11], and the generalized OR (GOR) (95% CI) [12].

## Results

### Identification of *RET *CDS mutations

In order to determine the mutational status of our whole cohort of 282 HSCR patients, we developed a mutational screening by dHPLC of the 176 cases that had not been analyzed yet. *RET *mutational status of remaining 106 cases was available from a previous study [7].

**Figure 1:**
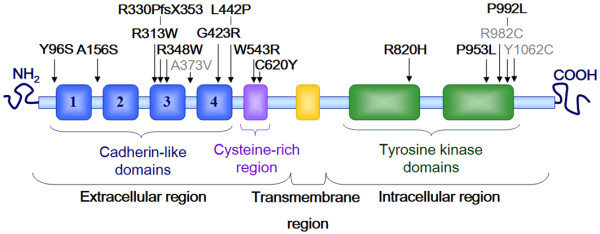
**Schematic representation of RET protein**. Mutations identified in this study are indicated in the corresponding functional domain. Novel and published mutations are presented in black and grey, respectively.

We have detected 16 different germline mutations in a total of 20 sporadic cases, all of them in heterozygosis (Table 1, Figure 1). 13 of the 16 mutations are novel and all of them, except for A156S, are predicted to be probably damaging variants using physical and comparative considerations http://genetics.bwh.harvard.edu/pph/ and http://blocks.fhcrc.org/sift/SIFT.html. Moreover, none of them were detected in the control population tested. 10/18 mutations are maternally inherited while inheritance from paternal origin occurs with a similar frequency than the *de novo *events (4/18 and 4/18 respectively). With this new data, our complete cohort of 282 HSCR patients presents a *RET *mutational frequency of 18.75% in familial cases (3 CDS mutations in 16 families screened) and 11.11% in non familial cases (29 CDS mutations in the 253 sporadic patients screened).

**Table 1:** *RET *germline mutations identified in our series of HSCR patients.

Patient	Exon/Intron	Nucleotide change	Amino acid change	Parent origin of the mutation	Gender	Length of aganglionic segment	**Ref**.
HSCR 193	2	c.287A > C	p.Tyr96Ser	Mother	Female	Not Available	--
HSCR 133	3	c.466 G > T	p.Ala156Ser	*De novo*	Female	S-HSCR	--
HSCR 166	5	c.937 C > T	p.Arg313Trp	Mother	Male	Not Available	--
HSCR V135	5	c.988insC	p.R330PfsX353	Mother	Male	S-HSCR	--
HSCR 223	5	c.1042 C > T	p.Arg348Trp	Mother	Male	Not Available	--
HSCR 109	6	c.1118 C > T	p.Ala373Val	Father	Female	S-HSCR	[7]
HSCR 217	6	c.1118 C > T	p.Ala373Val	Mother	Male	S-HSCR	[7]
HSCR 220	6	c.1118 C > T	p.Ala373Val	Mother	Male	S-HSCR	[7]
HSCR 99	7	c.1267G > A	p.Gly423Arg	Mother	Female	S-HSCR	--
HSCR V67	7	c.1325T > C	p. Leu442Pro	Mother	Male	Not Available	--
HSCR 187	8	c.1627 T > A	p.Trp543Arg	*De novo*	Female	S-HSCR	--
HSCR 260	10	c.1859G > A	p.Cys620Tyr	Father	Male	L-HSCR	--
HSCR V24	14	c.2459G > A	p. Arg820His	*De novo*	Male	Not Available	--
HSCR 204	17	c.2858 C > T	p.Pro953Leu	Mother	Male	S-HSCR	--
HSCR 278	18	c.2944 C > T	p.Arg982Cys	Not Available	Male	S-HSCR	[19]
HSCR V22	18	c.2944 C > T	p.Arg982Cys	Father	Male	Not Available	[19]
HSCR 15	18	c. 2975 C > T	p.Pro992Leu	*De novo*	Male	L-HSCR	--
HSCR 151	19	c.3185 A > G	p.Tyr1062Cys	Mother	Female	S-HSCR	[20]
HSCR 198	19	c.3185 A > G	p.Tyr1062Cys	Father	Male	Not Available	[20]
HSCR 278	20	c*4delTCTTinsAAA		NA	Male	S-HSCR	--

### Genotyping of *RET *SNPs

We have analyzed allelic and genotypic distribution of 3 *RET *SNPs for the new 150 sporadic HSCR cases not previously tested [4,8] and for 178 controls individuals. Allelic distributions for rs2435357 and rs2505532 showed similar statistical differences in cases vs controls than previously observed (χ^2 ^= 135.41 p < < 10^-6 ^and χ = 96.45, p < < 10^-6 ^respectively), [4,8]. No statistical difference was found for rs2565206, although a slight trend towards significance was observed (χ = 3.62, p = 0.0569263). However, when we performed this analysis, including not only the new cases but the whole series of sporadic patients, the difference in the distribution of this variant in cases vs controls get statistical significance (χ^2 ^= 6.74, p = 0.0094106). Haplotypic distribution also resulted statistically different when comparing HSCR patients with controls (χ^2 ^= 159.62 p < < 10^-6^). Moreover, TDT analysis performed for the 134 HSCR trios showed a statistical difference when comparing transmitted haplotypes *versus *non transmitted haplotypes (χ^2 ^= 87.82 p < < 10^-6^).

**Figure 2:**
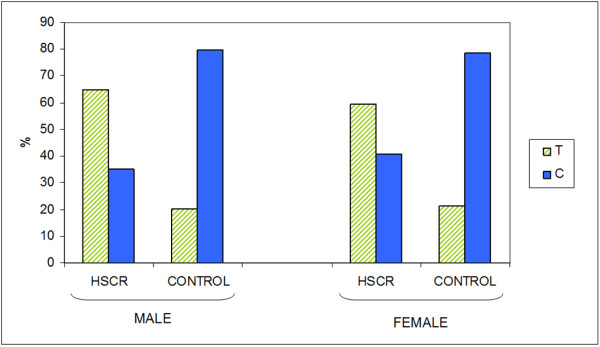
**Allelic distribution frequency of RET enhancer variant rs2435357 in Male and Female subset**.

On other hand, we have performed an additional statistical analysis comparing allelic, genotypic and haplotypic distribution in different subsets of patients and controls classified by gender (male patients vs male controls, and female patients vs female controls). Allelic distribution of rs2435357 and rs2505532 was clearly different in males and females HSCR patients compared with the corresponding control individuals, but presenting a considerably higher difference in male cases (23 orders of magnitude larger in males than in females, Table 2, Figure 2, Additional File 1). Regarding the rs2565206 variant, its distribution was found to be significantly different in male cases *versus *male controls whereas no difference was observed in female patients compared with female controls. Similar results were obtained for the analysis of the genotypic distribution. Haplotypic distribution was also different for both sets, although distribution was perceptibly more different in males (males: χ^2 ^= 167.6938, p = 2.26*10^-34 ^females: χ = 49.17491, p = 2.04*10^-9^). TDT applied to haplotypes was also performed in male and female subsets and resulted in an unequal transmission as we expected (males: χ = 114.5463, p = 4.47*10^-23^; females: χ = 42.91487, p = 3.84*10^-8^).

**Table 2:** Allelic distribution frequency of the *RET *SNPs

		Male		female	
***RET *variants**	**Alleles**	**HSCR (%)**	**Controls (%)**	**HSCR (%)**	**Controls (%)**

c.73+9277T > C	T	250 (64.77)	52 (20.15)	37 (56.06)	21 (21.43)
rs2435357	C	136 (35.24)	206 (79.85)	29 (43.93)	77 (78.57)
		**χ^2 ^= 121.79 p = 2.55·10^-28^←**		**χ^2 ^= 36.96 p = 0.0000117←**	

c.74-1370C > T	C	328 (84.97)	143 (55.43)	56 (84.85)	46 (46.94)
rs2505532	T	58 (15.03)	115 (44.57)	10 (15.15)	52 (53.06)
		**χ^2 ^= 67.22 p = 2.42·10^-16^←**		**χ^2 ^= 22.52 p = 0.0000021←**	

c.74-126G > T	G	300 (77.72)	175 (67.83)	48 (72.72)	70 (71.42)
rs2565206	T	86 (22.28)	83 (32.17)	18 (27.27)	28 (28.57)
		**χ^2 ^= 7.31 p = 0.0068453←**		χ^2 ^= 0.00 p = 0.9965511	

### Analysis of the relative position of *RET *CDS mutations and the enhancer variant

Both the genotyping of *RET *CVs and mutational screening of *RET *RVs was completed for 253 sporadic HSCR patients. Of them, 34 (13%) cases presented neither *RET *RVs nor the enhancer variant, 192 (76%) harbored only the enhancer variant in either heterozygosis or homozygosis, 13 (5%) had exclusively a *RET *RV (CDS), and 14 (6%) presented with both disease allelic types. The frequency distributions of these four mutational classes classified according to the gender presents similar results. Examining the patients with both kinds of mutational events, segregation analysis let us to observe that 91.66% of cases (11 of 12 patients) harbors the "enhancer mutation" *in trans *with respect the allele bearing the *RET *RV. The remaining patient was homozygous for the enhancer variant.

Of the 34 HSCR patients with no *RET *CDS mutation and no enhancer variation, 11 presented another kind of mutational events related with HSCR: 1 chromosomal translocation (t(10:22)), 6 chromosomal 21 triplication and 4 mutations in other HSCR genes (*EDNRB*, *GDNF*, and *NTF3*) [7,13,14].

## Discussion

Previous genome-wide linkage/association studies have identified several modifier genes in different genomic regions, but *RET *is the only gene known to play a major role in all forms of HSCR susceptibility and a quantitative study of its allelic spectrum should provide clues on the role of both rare and common sequence variants in the complex inheritance of HSCR, particularly its relationships to factors that are correlated with risk, such as gender.

After our *RET *CDS mutational screening, we have found that our whole series of Spanish HSCR patients presents a mutational frequency of 11.11% in sporadic cases and 18.75% in familial ones, which is concordant with previous studies that report values of up to 50% in familial forms and 7-20% in sporadic cases [2].

Genotyping analysis of *RET *common SNPs shows similar results to those previously described, confirming thereby the prominent role of either the intronic enhancer mutation (rs2435357, [3]) or the *RET *"risk haplotype" [4,8] in the pathogenesis of sporadic HSCR.

As previously commented, one of the major features of HSCR, especially in the short-segment forms, is the sex-dependent penetrance and male predominance of 4:1 [1]. Nevertheless the reason of this sex difference is still unclear. It has been postulated that sex differences could arise from mutations on the × chromosome, but genome-wide mapping studies have failed to identify an X-linked gene with a relevant impact *per se *in HSCR. Previous studies have indeed demonstrated differences in the transmission frequency of the "enhancer mutation" (rs2435357) depending on the offspring gender or parent gender [3,5]. For this reason, we sought to perform an additional case-control study to analyze the distribution of the *RET *variants based in gender. We found a different allelic distribution between HSCR cases and controls in both, males and females, for rs2435357 and rs2505532. However and noteworthy, significant differences in HSCR versus controls in the male subset were considerably increased with respect the female subset, which is concordant with the sex-biased transmission of the rs2435357 variant published by Emison et al. Nevertheless, no significant differences were found when comparing males vs females, in agreement with the similarity in transmission frequency by gender after applying TDT reported by Emison et al., 2010 [5]. The same phenomenon was observed for the haplotype analysis, which as a whole is consistent with the greater incidence in males than in females. However we cannot discard the possibility that the difference in the sample size of the corresponding subsets might be interfering to some extent in the results observed.

On the other hand, the association of rs2565206 to HSCR has been found to be sex-dependent and restricted to males. Probably, for this reason this SNP presented association to HSCR only when we extended our cohort, since we increased considerably the proportion of male HSCR cases. It is relevant to note that this specific variant has also been previously proposed to have a role in the pathogenesis of sporadic medullary thyroid cancer, since an over-representation of the T allele was found in different studies reported [8,15,16]. Although the most accepted hypothesis to explain these associations is the linkage with a still unidentified functional locus within or nearby *RET*, it has been proposed as well that this variant might slightly modulate *per se *the expression of the *RET *proto-oncogene, given that a new binding motif is created for NAFT transcription factor in the presence of the T allele [8]. The restriction to association of the G allele to the male subset of HSCR patients could be therefore considered as another hallmark of the complex nature of Hirschsprung disease.

After the evaluation of RVs and CVs in our HSCR series, we have classified our cohort of patients according to *RET *mutational status in 4 subsets (CDS mutation only, enhancer mutation only, both or any of those events) obtaining similar frequencies in these subsets than Emison et al [5]. Nevertheless, taking a further step in this study we performed a complete segregation analysis in patients with both RV and CV. 91.66% of cases with both mutational events resulted to present the enhancer variant in *trans *with regard to the CDS mutation. It supports that the CVs may act as modifier alleles of that bearing the RV, as speculated by Emison et al [5]. In this way, we could be talking about a synergy effect between two alleles, which is in agreement with the additive/multiplicative model proposed for HSCR.

On the other hand, in our cohort, we have detected a 13% of cases with no *RET *RVs or CVs. It has been previously proposed that mutations in other HSCR genes could be responsible for the HSCR phenotype in those patients, but the analysis of other HSCR genes and chromosomal alterations can only explain 31% of these cases. Alternatively, *RET *deletions have been also proposed as a possible underlying molecular mechanism [5], although our MLPA analysis revealed the absence of this kind of mutational event affecting *RET *or other HSCR genes [17,18]. Nevertheless, we cannot discard the presence of deletions in non-coding regulatory regions of *RET *or other HSCR genes which may lead to a loss-of-function of the protein.

## Conclusions

In summary, from our whole results we could extract several conclusions: (1) A gender effect exists on both the transmission and distribution of rare coding and common HSCR causing mutations (2) The variant rs2565206 has been found to be associated to HSCR in a sex-dependent manner, being such association restricted to male patients (3) *RET *CVs and RVs seem to act in a synergistic way leading to HSCR phenotype (4) A portion of HSCR cases without *RET *CVs and RVs might be explained by mutational events in another still unidentified HSCR loci, and efforts should be made to propose alternative methodological approaches that could lead to the elucidation of their molecular causes.

## Competing interests

The authors declare that they have no competing interests.

## Authors' contributions

SB coordinated all the studies here presented. JCA and LC recruited the HSCR patients and provided clinical information. RMF and SB developed the design of the experiments. RN-T, MJA and MVE-R carried out the molecular genetic analyses. MB performed the statistical tests. RN-T and SB drafted the manuscript. RMF and GA helped to draft the manuscript. All authors have read and approved the final manuscript.

## Pre-publication history

The pre-publication history for this paper can be accessed here:

http://www.biomedcentral.com/1471-2350/12/138/prepub

## Supplementary Material

Additional File 1 **Calculations of OR and GOR values**. OR (95% CI) and the generalized OR (GOR) (95% CI) were calculated for the recessive, dominant and additive models for each variant (unadjusted and adjusted for gender). Click here for file
