# Supplementary material for: Comprehensive analysis of RET common and rare variants in a series of Spanish Hirschsprung patients confirms a synergistic effect of both kinds of events
Source: BMC Med Genet. 2011 Oct 13;12:138. doi: 10.1186/1471-2350-12-138 (PMC3210088; doi:10.1186/1471-2350-12-138)
Supplement: Additional File 1 — Calculations of OR and GOR values. OR (95% CI) and the generalized OR (GOR) (95% CI) were calculated for the recessive, dominant and additive models for each variant (unadjusted and adjusted for gender). [file 1471-2350-12-138-S1.DOC]

|  |  |  | **OR** | 95% LL (OR) | 95% UL (OR) | **ORG** | 95% LL (GOR) | 95% UL (GOR) |  |
| --- | --- | --- | --- | --- | --- | --- | --- | --- | --- |
|  | c.73+9277T>C | additive.model | **60,9** | 7,230239 | 512,95818 | **60,89998** | 7,229957 | 512,9778 | |
|  | rs2435357 | recessive.model | **7,105** | 2,925962 | 17,25279 | **7,105** | 2,925915 | 17,25308 | |
|  |  | dominant.model | **26,52632** | 3,41236 | 206,20494 | **26,52631** | 3,412232 | 206,2125 | |
|  | c.74-1370C>T | additive.model | **20,8** | 4,985888 | 86,77291 | **20,8** | 4,985756 | 86,77522 | |
| Females | rs2505532 | recessive.model | **6,740741** | 1,794646 | 25,31842 | **6,740741** | 1,794602 | 25,31904 | |
|  |  | dominant.model | **17,018182** | 6,549236 | 44,22173 | **17,01818** | 6,549121 | 44,2225 | |
|  | c.74-126G>T | additive.model | **1,28** | 0,2909797 | 5,630634 | **1,28** | 0,2909718 | 5,630787 | |
|  | rs2565206 | recessive.model | **1,222222** | 0,2893433 | 5,16282 | **1,222222** | 0,2893356 | 5,162957 | |
|  |  | dominant.model | **1,137778** | 0,5326469 | 2,430387 | **1,137778** | 0,5326394 | 2,430421 | |
|  | c.73+9277T>C | additive.model | **31** | 13,09576 | 73,38253 | **31** | 13,09555 | 73,3837 | |
|  | rs2435357 | recessive.model | **8,14074** | 4,8781 | 13,58555 | **8,14074** | 4,878054 | 13,58568 | |
|  |  | dominant.model | **16,20857** | 7,193256 | 36,52279 | **16,20857** | 7,193148 | 36,52335 | |
|  | c.74-1370C>T | additive.model | **11,289474** | 4,699246 | 27,12185 | **11,28947** | 4,69917 | 27,12229 | |
| Males | rs2505532 | recessive.model | **5,285714** | 2,292831 | 12,18527 | **5,285715** | 2,292796 | 12,18546 | |
|  |  | dominant.model | **6,848947** | 4,166626 | 11,25805 | **6,848948** | 4,166588 | 11,25815 | |
|  | c.74-126G>T | additive.model | **3,8** | 1,45553 | 9,920782 | **3,8** | 1,455505 | 9,920957 | |
|  | rs2565206 | recessive.model | **3,234783** | 1,267895 | 8,252907 | **3,234783** | 1,267873 | 8,253051 | |
|  |  | dominant.model | **1,659494** | 1,058704 | 2,601218 | **1,659494** | 1,058695 | 2,601239 | |
|  | c.73+9277T>C | additive.model | **35,005435** | 15,81417 | 77,48624 | **35,00543** | 15,81394 | 77,48735 | |
|  | rs2435357 | recessive.model | **7,785284** | 5,00462 | 12,11094 | **7,785283** | 5,004579 | 12,11104 | |
|  |  | dominant.model | **17,554348** | 8,28208 | 37,20746 | **17,8125** | 8,40147 | 37,76545 | |
|  | c.74-1370C>T | additive.model | **13,38447** | 6,361906 | 28,15886 | **13,38447** | 6,361818 | 28,15924 | |
| All | rs2505532 | recessive.model | **5,749194** | 2,842327 | 11,62893 | **5,749194** | 2,84229 | 11,62908 | |
|  |  | dominant.model | **8,480191** | 5,467352 | 13,15329 | **8,48019** | 5,467307 | 13,15339 | |
|  | c.74-126G>T | additive.model | **2,810695** | 1,267569 | 6,232408 | **2,810695** | 1,26755 | 6,2325 | |
|  | rs2565206 | recessive.model | **2,464773** | 1,134095 | 5,356786 | **2,464773** | 1,134079 | 5,356863 | |
|  |  | dominant.model | **1,506992** | 1,024336 | 2,217072 | **1,506992** | 1,024328 | 2,217088 | |
